# Delivering balanced energy and protein (BEP) supplements to pregnant and breastfeeding women in low- and middle-income countries: A scoping review

**DOI:** 10.1371/journal.pgph.0007539

**Published:** 2026-11-02

**Authors:** Mihaela C. Kissell, Isabel Potani, Carolina Pereira, Elisabeth T. Mukendi, Vani Sethi, Filomena Gomes, Rimu Byadya, Lisa M. Rogers, Saskia J. M. Osendarp, Charity Zvandaziva, Saskia de Pee, Martin N. Mwangi

**Affiliations:** 1 Healthy Mothers Healthy Babies Consortium, Micronutrient Forum, Washington, District of Columbia, United States of America; 2 Independent Consultant, Ripple Global Health International, London, United Kingdom; 3 UNICEF Regional Office for South Asia, Kathmandu, Nepal; 4 NOVA Medical School, Universidade NOVA de Lisboa, Lisboa, Portugal; 5 Department of Nutrition and Food Safety, World Health Organization, Geneva, Switzerland; 6 Division of Human Nutrition and Health, Global Nutrition and Health Group, Wageningen University & Research, Wageningen, The Netherlands; 7 UNICEF, Lilongwe, Malawi; 8 Nutrition Division, World Food Programme HQ, Rome, Italy; Yale University, UNITED STATES OF AMERICA

## Abstract

In settings where more than 20% of women of reproductive age are classified as underweight, the World Health Organization recommends balanced energy and protein (BEP) supplementation during pregnancy to reduce adverse birth outcomes. However, guidance on contextually acceptable and feasible supplement delivery platforms remains limited, posing critical challenges for implementation. We synthesized literature on acceptable and feasible delivery platforms for BEP supplementation (direct evidence), as well as iron and folic acid (IFA) or multiple micronutrient supplements (MMS; indirect evidence) to pregnant and breastfeeding women (PBW) to broaden the search scope. A systematic search was conducted across five databases and grey literature to identify studies that addressed the acceptability and feasibility of BEP supplement delivery platforms in low- and middle-income countries (LMICs), at facility and community levels. We identified 48 studies conducted in LMICs: 22 specifically evaluated BEP supplement delivery, while 26 evaluated IFA or MMS. In the direct evidence, home delivery (11/22) was the most frequently mentioned delivery platforms of BEP supplements followed by community-level feeding centers (provided cooked meals or take-home rations) anchored by frontline community health workers (8/22 studies) and antenatal care (ANC) at health facilities (6/22; some studies assessed multiple delivery platforms). Only four studies assessed preferred BEP delivery platforms, and PBW favored home over facility-based delivery. Authors attributed this preference to the comprehensive nutrition education and behavior change communication (BCC) provided during home delivery. Indirect evidence showed that enhancing delivery of IFA or MMS at health facilities (not specific) through strategies such as BCC and home visits may improve supplement compliance. Overall, limited evidence exists regarding market-based approaches for delivering any of these interventions. Based on existing evidence, scale-up delivery of BEP supplementation through ANC at health centers, enhanced by community health workers may be feasible in reaching PBW, especially when combined with BCC.

## Introduction

Undernutrition during pregnancy is associated with adverse maternal and infant health outcomes, such as pre-eclampsia, stillbirth, and small-for-gestational-age (SGA) births [1]. To reduce the prevalence of stillbirths or SGAs, the World Health Organization (WHO) recommends providing balanced energy and protein (BEP) supplements to all pregnant women in settings with a high burden of undernutrition (≥ 20% underweight in women of reproductive age), rather than restricting supplementation only to individual participants defined as undernourished [2]. BEP supplements are commonly defined in the literature [3] as food products with varying macronutrient and micronutrient compositions aimed to improve the nutritional status of pregnant (and breastfeeding) women that provide less than 25% of energy from protein. Examples of BEP supplements include ready-to-use products such as lipid-based nutrient supplements, fortified flours such as corn-soya blends, or locally prepared foods or snacks such as prepared meals or eggs.

The WHO’s 2016 antenatal guideline provides limited operational guidance on how to deliver BEP supplements, specifically on which delivery platforms are most suitable [2]. Further, the WHO guideline remarks that the distribution of BEP supplements may not be feasible through the local health facility-based ANC clinics [2]. The guideline indicates it is essential to identify delivery platforms that are acceptable and feasible to support successful nutrition interventions [2]. This combination of a clear recommendation to provide BEP supplements and a lack of specific guidance on delivery platforms creates a critical implementation gap for countries considering scaling up. To address this gap, we proposed a pragmatic evidence synthesis drawing on multiple sources of nutritional supplementation evidence. There is still limited information about the most acceptable and feasible coverage platform to deliver BEP supplements to pregnant (and breastfeeding) women. BEP supplements have been implemented in far fewer settings than iron and folic acid (IFA) or multiple micronutrient supplements (MMS) but WHO recommends all these interventions to be delivered in the antenatal care (ANC) settings [2,4]; thus, we treated IFA and MMS delivery evidence as informative in generating hypotheses about delivery and implementation of BEP supplementation where direct data were sparse. The objective of this work is to summarize direct and indirect evidence on the acceptability and feasibility of delivery platforms for BEP supplementation among pregnant (and breastfeeding) women in low- and middle-income countries (LMICs) to inform future BEP supplement programming.

## Methods

This scoping review was conducted in accordance with Preferred Reporting Items for Systematic Reviews and Meta-Analyses extension for Scoping Reviews (PRISMA-ScR) guidelines [5]. The PRISMA-ScR checklist is provided as S1 Checklist.

### Search strategy

In this scoping review, a comprehensive literature search (S1 Table) was conducted with the assistance of a research librarian across five electronic databases (Cochrane Library, PubMed, Embase, CINAHL, and Web of Science), covering the period from inception until October 01, 2024, and updated on April 13, 2026. We used broad search terms, including keywords related to food supplementation in pregnancy and lactation/breastfeeding, to identify as many relevant articles as possible. The searches had no date, language, or other restrictions.

### Inclusion and exclusion criteria

We included primary studies reporting on delivery platforms outcomes of BEP supplementation, IFA, or MMS because WHO recommends all to be delivered in the ANC context [2,4] and to inform hypotheses about delivery and implementation of BEP supplementation among pregnant and breastfeeding women in LMICs. We included only articles published in English. We considered BEP supplements, as defined in the Introduction section. IFA is a prenatal supplement containing iron and folic acid. MMS was defined according to WHO: a prenatal supplement containing at least three micronutrients in addition to iron and folic acid [4]. We excluded review articles, protocols, clinical trial registries, or conference abstracts, studies conducted in high-income countries, and non-human studies.

### Data extraction

We screened titles and abstracts of the articles, followed by the full-texts screening for eligibility in Covidence [6] and Excel. At both screening stages, each study was screened in duplicate by any two of the four reviewers (IP, EM, CP, and MCK), who screened independently of each other. Disagreements were resolved by a third reviewer (EM, CP, or MCK) who had not been involved in the initial screening of the disputed article. From the eligible studies, we extracted the study author, year of publication, study design, aim, supplement tested, population, region, and delivery platforms information.

### Data synthesis and analysis

Outcomes were categorized as direct evidence if they came from studies specifically addressing BEP supplementation, and as indirect evidence if they came from IFA or MMS studies. Within each evidence category, delivery platforms were identified, and country-specific platforms were outlined. The findings were summarized and presented in a table.

## Results

The literature search identified 4,986 articles for screening (Fig 1). After removing duplicates and ineligible studies, 48 eligible studies were included in this review [7–53], of which 22 focused on BEP supplementation (direct evidence) and 26 focused on IFA and MMS (indirect evidence detailed in Results in S1 Appendix and corresponding S2 Table). The eligible studies were conducted among pregnant and breastfeeding women and assessed outcomes of delivery platforms related to health facilities, feeding centers, markets/pharmacies, and home delivery of supplements in LMICs (Table 1, S2 Table). The study designs included feasibility studies, randomized controlled trials (RCT), and mixed-methods quasi-experimental studies. The included primary studies (n = 22) analyzed data using quantitative and qualitative approaches to assess the acceptability and feasibility of the various delivery platforms.

**Table 1:** Description of included literature on balanced energy and protein (BEP) supplement delivery platforms in low- and middle-income countries.

Author	Study design/data collection type	Country	Objective	Population at enrolment	BEP supplement form	Delivery platform reported results
Kalbarczyk et al., 2024 [9]	Formative study – qualitative methods	Bangladesh	To assess implementation outcomes for launching BEP supplementation	95 married women of reproductive age (some were pregnant and some had recently given birth),family members,health care providers,pharmacists	Not specified	Perceptions for home delivery, ANC clinic, and pharmacy delivery
Novak et al., 2006 [16]	Pilot study – qualitative methods	Bangladesh	To evaluate food supplementation, nutritional intake of recipients, and operational aspects of an integrated pilot nutrition initiative	Unspecified number of pregnant and breastfeeding mothers, adolescent girls in school (11–18 years old) and children below 2 years of age	Boiled, roasted flat rice, peanuts, and molasses rolled into balls	Home delivery
Kok et al., 2021 [17]	Randomized cross‐over study – mixed methods	Burkina Faso	To evaluate home consumption of two fortified BEP supplements by pregnant women	A total of 80 pregnant women aged between 15 and 40 years	LNS peanut paste,vanilla biscuit	Home delivery
Janmohamed et al., 2016 [7]	Explanatory sequential study - qualitative methods	Cambodia	To identify factors affecting the acceptability and consumption of Corn Soya Blend Plus (CSB+) as a prenatal supplement	A total of 288 pregnant women for the structured interviews and 70 pregnant women to further explore attitudes, perceptions, and experiences related to CSB + use	(CSB+) Flour	Home delivery and health post collection
Eglovitch et al., 2024 [14]	Prospective pilot study - qualitative methods	Ethiopia	To assess the adherence and acceptability of BEP supplementation among pregnant women	A total of 40 pregnant women	(CSB+) Flour	Health post or home delivery, depending on the participant’s preference
Lama et al., 2022 [15]	Formative research (phase 1)- qualitative methods	Nepal	To assess compliance with and acceptability of savory and sweet fortified supplements among pregnant women	A total of 40 pregnant women	Soup, sticks, bars, paste, biscuits, drinks	Home delivery and willingness to buy
Lama et al., 2022 [18]	Formative research (phase 2) -mixed-methods	Nepal	To assess and compare compliance with and acceptability of two BEP supplements, and to identify BEP supplements for subsequent inclusion in an efficacy trial	A total of 80 pregnant women	LNS peanut paste, and biscuit	Purchasing from markets
Isanaka et al., 2019 [19]	Two-part, multi-methods study - qualitative	Niger	To determine the acceptability and utilization of a lipid-based nutrient supplement formulated for pregnant women	A total of 25 pregnant women aged ≥ 18 years	MQ-LNS	ANC delivery and willingness to buy
Clermont et al., 2018 [13]	Two-faced longitudinal study with emergent design (phase 2) - qualitative methods	Niger	To evaluate the acceptability and utilization of three nutritional supplements during pregnancy	Pregnant women (84 interviews and 17 FGDs discussion in phase 1; 39 interviews and 12 FGDs in phase 2), household members, and study staff	MQ-LNS	Home delivery
Adams et al., 2018 [25]	Longitudinal willingness-to-pay data from an RCT- mixed methods	Ghana and Malawi	To characterize private valuation of SQ‐LNS over the course of recommended consumption and, using a diverse set of covariates, explore the factors associated with willingness to pay	A total of 792 and 626 pregnant women (<20 weeks of gestation) in Ghana and Malawi, respectively	SQ-LNS	Purchasing from markets
Ramakrishnan et al., 2012 [24]	Cross-sectional study - mixed methods	India (with a focus on 2 states: Tamil Nadu and Uttar Pradesh)	To evaluate the implementation of maternal nutrition programs in India	Pregnant women and breastfeeding women (diverse respondents)	Cooked food	Community feeding centers and home delivery
Singh et al., 2020 [47]	Nationally representative cross-sectional study – quantitative methods	India	To examine the socioeconomic, programmatic, and contextual factors associated with the consumption of IFA tablets/syrup for at least 100 days (IFA100) and receiving supplementary food (SF) by pregnant women in India	A total of 190,898 ever-married women aged 15–49 years who had at least one birth 5 years preceding the survey	Cooked meals or take-home rations	Community feeding centers
Bhatnagar et al., 1982 [26]	Prospective cohort study – quantitative methods	India	To study the weight gained by the mothers receiving defined supplementary nutrition and to compare it with the weight gain of a matched group of mothers not receiving the additional nutrition, and to compare birthweight and growth patterns of the infants of the two groups of mothers up to 3 months of age	Pregnant women (24 weeks of gestation)	Supplementary food: local recipe providing 16 g of protein, 300 kcals	Integrated Child Development Services (community feeding center) vs. non-Integrated Child Development Services women
Chudasama et al., 2016 [21]	Cross-sectional program evaluation study– quantitative methods	India	To evaluate the various aspects of the ICDS program in terms of inputs, process, and outcome (coverage), utilization, and issues related to the ICDS program	A total of 130 Anganwadi centers (95 rural and 35 urban)	Cooked food or take-home ration	Community feeding center
Hoque et al., 2010 [22]	Cross-sectional observational study – quantitative methods	Bangladesh	To determine the compliance to intake of food and micronutrient supplements among 48 rural pregnant women in two sub-districts under two divisions of Bangladesh	A total of 48 rural pregnant women	Cooked food	Communityfeeding centers
Coreil, 1987 [27]	Cross-sectional survey study – quantitative methods	Haiti	To monitor Maternal and Child Health Feeding Centers that provided Title II supplementary feeding programme	A total of 39 centers out of 519	Cooked or uncooked food	Community feeding centers
Sethi et al., 2019 [23]	Cross-sectional program evaluationstudy – mixed methods	India	To evaluate the maternal spot feeding programs in 2 Southern Indian states	A total of 360 pregnant and breastfeeding women (of infants aged 0–6 months)	Cooked food	Community feeding centers
Ashraf et al., 2025 [50]	Cross-sectional survey, quasi-experimental design – quantitative methods	Pakistan	To evaluate the effectiveness of a nutritional supplementation programme, underpinned with behavior change communication and implemented through Lady Health Workers	2,821 and 2,410 pregnant and lactating women at baseline and endline, respectively	Wheat Soya Blend (WSB)	Home delivery by CHW
Dhasmana et al., 2025 [48]	Exploratory study -qualitative methods	India	Explored opportunities to enhance dietary diversity and environmental sustainability within the Supplementary Nutrition Program under ICDS in Andhra Pradesh	Pregnant and lactating women but number not indicated	Take-home ration	Community feeding centers
Dolati et al., 2025 [49]	Retrospective field trial study – mixed methods	Iran	To compare the effects of the food basket program and the cash transfer program to support pregnant and lactating mothers in needy suffering from malnutrition in Iran.	1,331 pregnant and lactating women	Food basket and cash transfer	Health center and market delivery
Nguyen et al., 2024 [52]	Instrument development stud and cross-sectional survey study - mixed methods	India	To 1) develop and cognitively test household survey questions that measure coverage of nutrition‐sensitive social protection programs in India; and 2) use these questions to estimate the coverage of nutrition‐sensitive social protection programs and co‐coverage with other nutrition‐relevant interventions	6,627 mothers	1. reconstitutable food supplements; 2. grains and/or lentils; 3. cooked food; 4. cash	Market delivery (purchased food from ration shops); home delivery and cash (bank) transfers
Shifraw et al., 2026 [51]	Formative study – mixed methods	Ethiopia	Assessed the acceptability, adherence, and sharing practices of a ready-to-use BEP supplement among pregnant women in Addis Ababa, Ethiopia	45 pregnant women	MQ-LNS	Home delivery

BEP – Balanced energy and protein supplementation; RCT – Randomized controlled trial; FGD – Focus group discussion; CSB + – Corn Soya Blend Plus; LNS – Lipid-based nutrient supplement; ANC – Antenatal care; IFA – Iron and folic acid; MMS – Multiple micronutrient supplement; MQ-LNS – Medium-quantity lipid-based nutrient supplement; SQ-LNS – Small-quantity lipid-based nutrient supplement; ICDS – Integrated Child Development Services; N/A – Not applicable.

**Fig 1:**
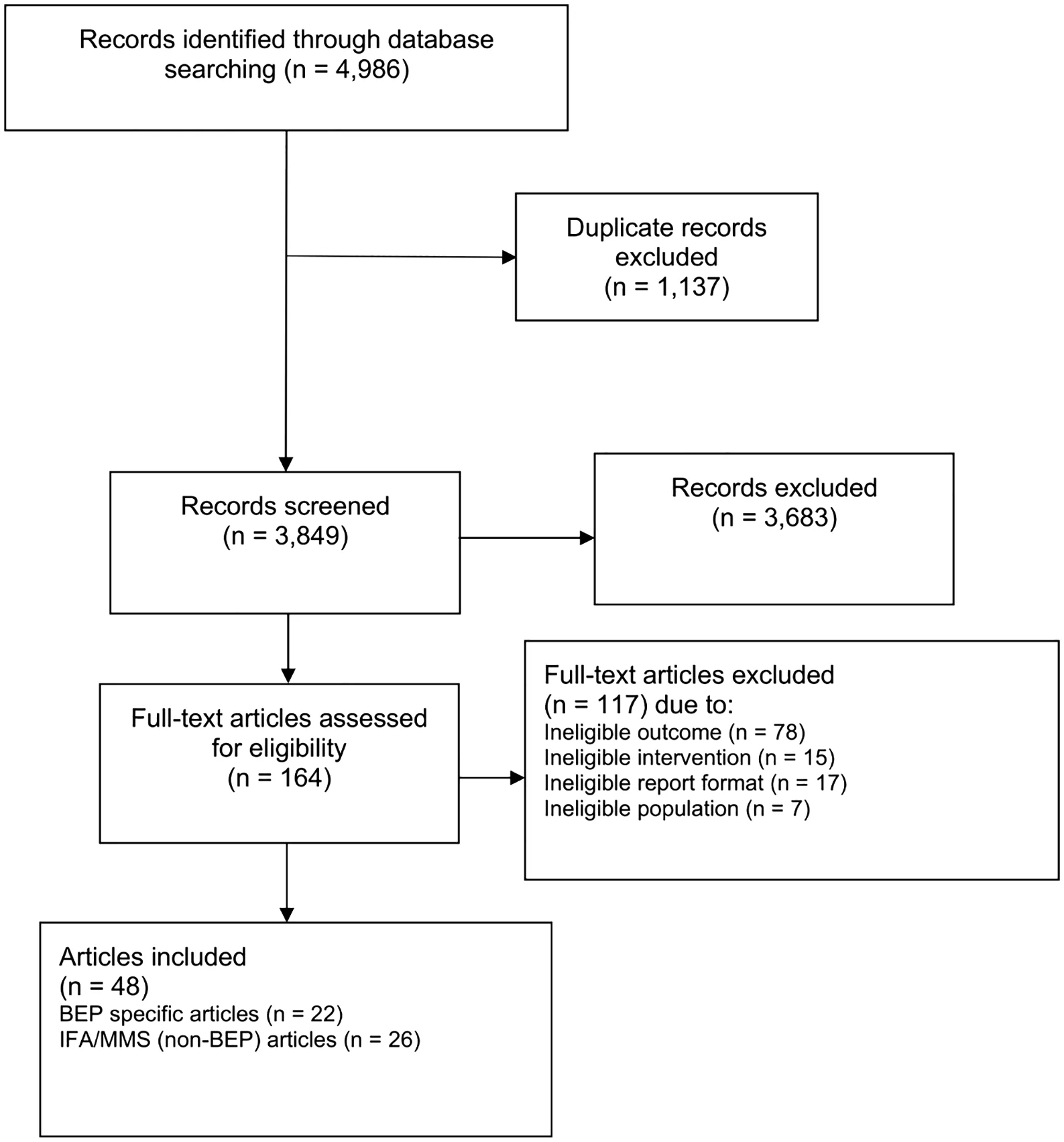
Prisma flow chart of study selection in a scoping review on balanced energy and protein supplementation. Abbreviations: BEP = Balanced energy and protein dietary supplementation; IFA = Iron and folic acid; MMS = Multiple Micronutrient Supplements.

### Direct evidence on BEP supplements

There were 22 studies that provided direct evidence on BEP supplement delivery platforms [7,9,13–22,24–27,47–52]. BEP supplementation was delivered in diverse forms across studies, including ready-to-eat products (e.g., lipid-based nutrient supplements (LNS) or biscuits), fortified-blended foods or cereals (e.g., Corn-Soya Blend Plus (CSB+)), cooked meals, or meal ingredients (e.g., eggs). In South Asia, programs frequently provided either dry take-home rations (providing eggs, milk, locally made snacks, etc.) or freshly cooked meals, community/spot feeding programs, both under India’s supplementary nutrition program called Integrated Child Development Services (ICDS) [21,24,26,48,52]. Some studies were exploratory and did not specify the BEP supplement form; they explored the effects, coverage, access of nutritious foods during pregnancy and breastfeeding without specifying the precise form [9,49,52].

### Community feeding centers of BEP supplements

Community feeding centers were used as a primary BEP supplement delivery platform in eight of the 22 BEP supplementation studies identified, conducted in India, Bangladesh, and Haiti [21–24,26,27,47,48]. One of two studies on community feeding centers in Bangladesh (n = 48 pregnant women) reported that 16% of the participants consumed the supplement alone at the community feeding centers, while 24% shared it with other women there [22]. At home, 17% took the supplement alone and 9% shared it with family members. Additionally, 16% took the supplement home but did not consume it during the observation period, and 17% did not visit the centers to receive the supplement. Study authors speculated that the reasons for low attendance to collect BEP supplements were “due to a lack of cooperation by the Centre staff or lack of knowledge by both the mothers and the staff regarding supplements” [22]. In the second study from Bangladesh, which also examined BEP supplementation at feeding centers, mothers consumed about 75% of the food provided, and that the remainder of the food was shared mostly with young children, husbands, or other family members [16]. The study found that sharing was more common in larger households. It also noted that, although nutrition education was part of the original intervention design, it was not delivered alongside the BEP supplementation. The authors speculated that this lack of educational content may have led to increased sharing of the BEP supplements [16]. Similarly, in India, across Anganwadi community centers, inconsistency in training of community health workers and the need for cooks to cook for many people to improve meal acceptability, posed challenges [48]. Additional challenges reported include delays in receiving food ingredients and reimbursements, inadequate storage conditions, challenges in using web applications for food distribution, and fluctuations in food costs [48]. In turn, these challenges led to inadequate program implementation and food quality [21,24]. Authors suggested promoting service integration, ensuring effective procurement platforms, establishing regional training facilities to improve program implementation, and strengthening program monitoring and evaluation could improve the challenges [24]. Sethi et al. reported that the enrollment in self-help groups and family members in India motivated 68% of pregnant and breastfeeding women to attend feeding centers; these delivery platforms enhanced maternal nutrition service quality and quantity [23]. Dhasmana et al. found that program workers find monthly nutrition education and skill-based training sessions helpful and important in providing good nutrition behavior such as consumption of fortified rice, which beneficiaries viewed as “plastic rice” due to its lighter texture [48]. In Haiti, a privately sponsored supplementary feeding program reported that postnatal nutrition supplementation was primarily used to incentivize women to attend family planning services [27].

### Home delivery of BEP supplements

Eleven of the 22 included BEP supplementation studies [7,9,13–17,24,50–52] conducted home delivery. For instance, in Bangladesh, Niger, Ethiopia, and Cambodia, some participants were receptive to home delivery of BEP supplements [7,9,13,14]. In Niger, health assistants reported that the supplements were well accepted by participants, who found the weekly home visits helpful and convenient [13]. In addition to BEP supplement delivery, participants indicated the health assistants played a critical role in sensitizing them and their families to health topics. Because many of the health assistants were male, this was initially met with resistance from participants’ husbands; however, study awareness campaigns were able to overcome this resistance. Similarly, in Cambodia, 99% of participants reported in interviews that if the supplement (CSB+ form) were free of cost, home delivery would be the preferred platform (as done during the study) rather than picking it up from the health center [7]. In an acceptability study conducted in Bangladesh, community health workers reported that delivering BEP supplements (in an unspecified form) to participants’ homes enables them to provide comprehensive nutrition counseling; however, there is a need for more community health workers and higher pay for these services [9]. In contrast, one study in rural Pakistan reported challenges with providing BEP supplements (wheat-soya blend form) in participants homes by community health workers due to an interrupted supply chain, less supplement prioritization, and sharing of the supplement with households. The solution suggested was to conduct quality behavior change communication that address barriers and enablers through nutrition education [50].

### Health facility delivery of BEP supplements

Integration of BEP supplement delivery within ANC at health facilities occurred in six studies conducted in Niger, Ethiopia, Burkina Faso, Cambodia, India, and Iran [7,9,14,17,19,49]. Notably, the Iranian study evaluated a national program that provided two types of interventions, one that provided BEP supplementation (food baskets) at the health center and another one that provided cash transfers via banks aimed at purchasing nutritious foods; all beneficiaries received health and nutrition education [49]. In Ethiopia, 74% participants indicated that they would like to receive the BEP supplement (CSB form) at a health center or community health post but did not specify the reason [14]. In this study, BEP supplements were initially provided at health centers at enrolment, and then the supplements were provided according to participants’ preference at subsequent visits either at home or health center. In a Cambodian study, 80% of participants reported being happy to collect the BEP supplement (CSB form) from the health center; notably, the study protocol was to deliver the BEP supplement to participants’ homes [7]. In Bangladesh, participants expressed concerns about the acceptability of integrating BEP supplementation into ANC services. Although clinics were viewed as a feasible distribution point due to their wide availability and participants’ familiarity with attending them on scheduled days, many described them as overcrowded, of low quality, and affected by perceived corruption and diversion of medicines. Both participants and healthcare providers noted that distance to clinics reduced attendance and access to ANC counselling. Nonetheless, providers acknowledged that clinics offered advantages for delivering information directly to participants during their visits [9]. Notably, in Burkina Faso, recipients, health providers, and policymakers reported that health facilities are the best places for delivering supplements, even though the BEP supplement was delivered at home in the study [17].

### Market delivery of BEP supplements

Seven studies conducted in Ghana, Malawi, Bangladesh, Iran, India, Niger, and Nepal explored market-based approaches, (i.e., purchasing or selling supplements at full cost, unless otherwise specified) from local markets or pharmacies [9,15,18,19,25,49,52]. As noted above, the Iranian study evaluated participants enrolled in national programs that either provided BEP supplements in the form of food baskets through health centers or cash transfers (USD 21 every two months) intended to purchase nutritious foods [49]. However, the study did not monitor what participants purchased with the cash transfers. Both programs also included health and nutrition education on beneficial foods during pregnancy and breastfeeding periods. The study did not report whether participants preferred receiving support through the health centers or cash transfers to bank accounts [49]. Another study from India reported two transfer modalities within national supplementary programs: food transfers and cash transfers [52]. Food transfers included reconstituted food supplements, grains and/or lentils, and cooked food often delivered at the community level by community health workers, while cash was transferred directly to bank accounts. However, the study provided limited details on the nutritional composition of the transferred foods and the food items purchased with the cash transfers [52].

There were mixed findings on the purchasing behavior of BEP supplements by pregnant and breastfeeding women [15,18,19,25]. In rural Nepal, one study found that participants would purchase BEP supplements (in the LNS form; 72–75 g ration), if they were available on the market [18] whereas others said they could not afford them [15]. Similarly, in Bangladesh, local pharmacies were described as a convenient and trusted channel for distributing BEP supplements (75 g ration). However, potential conflicts with existing commercially promoted products discouraged pharmacists from supporting BEP supplements distribution unless incentive structures were realigned [9]. Qualitative findings from rural Niger indicated that participants expressed willingness to pay on average USD 0.31 for BEP supplements in the form of LNS (40 g ration), which is equivalent to the average of amount last paid of one serving of favorite snack food [19]. Similarly, quantitative findings from a study conducted in Ghana showed that participants were willing to pay for LNS (20 g ration), for up to USD 0.66 (daily supply), whereas in Malawi, participants were willing to pay about USD 0.90 (weekly supply) [25]. In Ghana, willingness to pay for LNS was influenced by time since enrolment, respondent type (mother vs. head of household), household food security, and maternal morbidity. In Malawi, willingness to pay was associated with time since enrolment, seasonal food availability, and household food security. Specifically, in Ghana, willingness to pay declined with each additional month since enrolment (−USD 0.03/month, p = .001) and was lower among mothers than among heads of household (−USD 0.15/month, p = .01; − USD 0.08 long-term, p = .05). Household food insecurity was significantly associated with reduced willingness to pay, and mothers experiencing nausea or vomiting reported lower willingness to pay (−USD 0.08/day, p = .05; − USD 0.09 long-term, p = .004). Other morbidity indicators, such as poor appetite and diarrhea, were positive but not significantly associated with willingness to pay. In Malawi, willingness to pay increased with time since enrolment (+USD 0.03/month, p = .05). In both Malawi and Ghana, household food insecurity was negatively associated with willingness to pay (p = .03) [25].

### Indirect evidence on IFA and MMS supplements

A total of 26 studies evaluated supplementation with IFA or MMS without any BEP supplementation [11,20, 28–45, 53–58] as shown in Results in S1 Appendix and corresponding S2 Table. Twenty-one studies examined IFA supplementation alone across LMICs, particularly in South Asia and sub-Saharan Africa. Three additional studies combined IFA with calcium supplementation and three studies focused on MMS. Health facility delivery was the most reported platform (17 studies), followed by home delivery (4 studies); one study reported on community feeding centers and another one on market delivery platform. This evidence is further described in the Results in S1 Appendix and corresponding S2 Table. Overall, this indirect evidence showed mixed findings regarding ANC health facility delivery, home delivery (including direct observation of intake), and market delivery approaches. However, an important conclusion emerged, enhancing IFA or MMS delivery at health facilities (not specific) through strategies such as behavior change communication and home visits or community mobilization, may improve adherence.

## Discussion

This scoping review summarizes direct and indirect evidence on the acceptability and feasibility of delivery platforms for BEP supplementation among pregnant (and breastfeeding) women from LMICs to inform future BEP supplementation programing. We found that hybrid facility-based BEP supplement delivery approaches, incorporating enhancements such as home deliveries and behavior change communication with health facility-based ANC services, are acceptable and may enhance supplement adherence. Despite the variations in the acceptability of BEP supplementation delivery platforms, home delivery was preferred over health facilities in most countries. The main reason was the convenience of home deliveries. Nevertheless, there were rare instances in which, Ethiopian participants preferred health facility to home delivery (74%). Further, if home delivery occurred, they indicated that they would prefer receiving the supplements twice per month rather than weekly (63%); however, details were not provided for this preference [59].

Given the limited evidence on the feasibility of BEP supplementation home delivery in non-study conditions, testing the feasibility of home-delivered food supplements among pregnant and breastfeeding women before implementation while considering product form, preparation type, and weight, transportation and distribution (including frequency of delivery), storage, cost, and household sharing of these supplements would be worthwhile in each setting. Delivery approaches that bring BEP supplementation closer to the homes of pregnant and breastfeeding women may particularly improve access to adequate nutrition in remote or marginalized communities [60].

Studies document community health workers provide supplementary or therapeutic foods to undernourished children at community health posts in Africa. Expanding these services to eligible pregnant and breastfeeding women and not only those treated at supplementary feeding programs may improve their nutritional status [61–63]. Although these are not home deliveries to pregnant and breastfeeding women, these delivery points are closer to the recipients’ (children with undernutrition) homes. The indirect evidence from IFA and MMS (Results in S1 Appendix and corresponding S2 Table) also supports this model of enhancing health facility delivery with home delivery and/or community mobilization in promoting supplement adherence [29,32,37,40,43,58]. Health posts rather than health centers could facilitate the delivery of BEP supplementation interventions for pregnant or breastfeeding women as well. However, the feasibility of this hypothesis (i.e., using community health workers to deliver BEP supplements to beneficiaries) would need to be tested before implementation in ANC.

We postulate that community-based delivery (including home or health post-delivery) of the BEP supplement may be as effective as health facility delivery in improving nutrition outcomes for pregnant and breastfeeding women, based on evidence from children. For instance, the proportion of children recovered from undernutrition in community-based deliveries of supplementary or therapeutic food supplements is similar to that in facility-based deliveries [61,62,64]. However, there are concerns about the home delivery of BEP supplements by community health workers, including inadequate skilled human resources (such as knowledgeable community health workers) and a lack of safe storage (due to pests and rodents, and inadequate temperatures and ventilation) for the supplements [48,63,65]. This aspect was also noted in the indirect evidence on IFA and MMS (Results in S1 Appendix and corresponding S2 Table) and in some settings community health workers were limited to providing only nutrition counselling [57]. Notably, in 2018, WHO recognized the potential of community health workers in delivering health interventions but highlighted the need for supportive systems to ensure effective health service delivery [66]. Specific to therapeutic food distribution, the 2023 WHO guideline on managing acute malnutrition in children allows community health workers to treat undernutrition, but they need to receive adequate training and supervision [66]. WHO could consider recommending individual screening of women with undernutrition, in addition to population-level screening when resources are limited.

Although some pregnant and breastfeeding women reported willingness to pay BEP supplements in the form of LNS, no published studies have tested this mode of delivery instead, participants were only asked about their willingness to pay for these supplements. Notably, two studies that tested willingness to pay for BEP supplements in LNS form should be interpreted with caution. These supplements provide 237 kcals and 5.2 g of protein for a 40 g ration [19] and 118 kcals and 3 g of protein for a 20 g ration [25], respectively, which although the energy provided from protein is still less than 25% (per definition of BEP supplements), the total energy and protein may not be enough to cover the energy needs of pregnant or breastfeeding women. In addition, the studies that provided food baskets, food supplements, grains, and cash transfers did not report on the nutrition composition of these foods or what participants purchased; as such, we are not certain if they would qualify as BEP supplements. Separately, based on the indirect evidence on IFA and MMS (Results in S1 Appendix and corresponding S2 Table), an ongoing trial aims to assess the effectiveness of delivering IFA supplements through market-based approaches and will provide more insights on the purchasing behavior of pregnant women [10]. Nevertheless, there is still a need for more studies investigating the purchasing behavior of pregnant and breastfeeding women, specifically for food supplements like BEP supplements, rather than only tablets like IFA or MMS, across different factors such as price points and affordability.

Key strengths of this scoping review include its comprehensiveness, recency, and novelty in focusing on BEP supplements. A similar review previously conducted assessed the evidence of community-based distribution of maternal nutrition supplements in LMICs and emphasized coverage and adherence [67]. A notable difference from the earlier review is that it identified only four studies, whereas we identified 22 (plus 26 studies focused on IFA and MMS), indicating a broader, more diverse evidence base. Another similar review, which focused solely on IFA supplementation, concluded that community-based platforms, such as door-to-door delivery and health posts, improved uptake and compliance [68]. Notably, some of the included studies recruited children, men, and women of reproductive age. Home delivery was implemented by community health workers and community volunteers. Most of the literature on the home distribution of IFA in earlier reviews was from South Asia, where community health workers provided IFA supplementation at home. In their narrative review, Kavle and Landry [68] concluded that community-based platforms, such as door-to-door delivery and health posts, improved uptake and compliance [68]. A key limitation of this scoping review is the lack of direct evidence on the acceptability and feasibility of different BEP supplementation delivery platforms given in the ANC context, especially along co-interventions. We also evaluated the IFA and MMS indirect evidence to inform hypotheses on delivery mechanisms for BEP supplementation. However, the delivery platforms for IFA and MMS are not directly comparable to BEP supplementation delivery platforms for pregnant and breastfeeding women because BEP supplements tend to be heavier and bulkier, differ in form and preparation, are costlier and more vulnerable to damage during storage and transport. In addition, they are often shared with household members unlike tablets like MMS and IFA.

## Conclusion

Across LMIC settings, BEP supplements reached pregnant and breastfeeding participants through several platforms, community feeding centers, home delivery, health facility, and market delivery. However, two patterns stood out: home delivery, either as the primary channel or as an enhanced ANC model that pairs routine health facility distribution with home visits, behavior-change counseling, directly observed intake, or social mobilization. The latter delivery platform appears more promising than health facility-only delivery. Next, community feeding centers were primarily used in South Asia, and evidence of market-based approaches remains scarce. Given substantial heterogeneity in health-system capacity, geography, and social norms, no single delivery platform is universally optimal. In addition, future studies should assess the requirements to include BEP supplements to the existing ANC services provided and the most feasible platform. Policymakers and implementers may consider approaches that adopt a flexible, context-specific, and evidence-driven approach that combines facility- and community-based delivery to strengthen reach and adherence. National programs are encouraged to embed formative research into planning cycles to identify context-specific delivery barriers and enablers, and to commission pragmatic evaluations. Evidence from such evaluations should inform adaptive policy design and sustainable scale-up strategies.

## Supporting Information

S1 Checklist Preferred Reporting Items for Systematic reviews and Meta-Analyses extension for Scoping Reviews (PRISMA-ScR) Checklist. (DOCX)

S1 Table Web of Science Search Strategy for Balanced Energy and Protein (BEP) Supplementation Delivery Platforms: Scoping Review. (DOCX)

S2 Table Included Studies on Delivery Platforms for Iron and Folic Acid, and Multiple Micronutrient Supplements Among Pregnant and Breastfeeding Women. (DOCX)

S1 Appendix Indirect Evidence on IFA and MMS Supplementation. (DOCX)
