# Supplementary material for: Delivering balanced energy and protein (BEP) supplements to pregnant and breastfeeding women in low- and middle-income countries: A scoping review
Source: PLOS Glob Public Health. 2026 Nov 2;6(11):e0007539. doi: 10.1371/journal.pgph.0007539 (PMC13726957; doi:10.1371/journal.pgph.0007539)
Supplement: Supplementary file 2 [file pgph.0007539.s002.docx]

# **S1 Table:** Web of Science Search Strategy for Balanced Energy and Protein (BEP) Supplementation Delivery Platforms: Scoping Review

| # | Search Query |
| --- | --- |
| 1 | TS=( "balanced energy protein supplements" OR "energy protein supplements" OR "protein-energy supplements" OR "dietary protein supplements" OR "balanced energy-protein" OR "balanced energy and protein" OR "balanced protein-energy" OR "protein supplement" OR "energy supplement" OR "lipid supplement*" OR "lipid nutrient*" OR "lipid spread*" OR "lipid paste*" OR "nutributter" OR "plumpy" OR "fortified blended food" OR "specialized nutritious food" OR "ready to use food" OR "corn soy* blend" OR "wheat soya blend" OR "super cereal" OR "high energy biscuit" OR "MAMTA" OR "mumta" OR "food assistance" OR "food distribution" OR "food specialized" OR "food aid" OR "food ration" OR "food supplement*" OR "food basket" OR "supplement food" OR "supplement feeding" OR "oral supplements" OR "fortified foods" OR "powder mixes" OR "protein bars" ) |
| 2 | TS=("women" OR "pregnant women" OR "mothers" OR "females" OR "prenatal" "perinatal" OR "antenatal") |
| 3 | TS=(("low-income countr*" OR "middle-income countr*" OR "developing countr*" OR "low-resource countr*" OR "resource-poor countr*" OR "underdeveloped countr*" OR "less developed countr*" OR "global south" OR "sub-Saharan Africa" OR "Latin America" OR "South Asia" OR "Southeast Asia" OR "Central America" OR "Eastern Europe" OR "Caribbean" OR "Pacific Islands" OR Afghanistan OR Angola OR Bangladesh OR Benin OR Bhutan OR Bolivia OR Botswana OR Burkina Faso OR Burundi OR Cambodia OR Cameroon OR Central African Republic OR Chad OR Comoros OR Congo OR "Democratic Republic of the Congo" OR Djibouti OR Egypt OR El Salvador OR Eritrea OR Eswatini OR Ethiopia OR Fiji OR Gambia OR Ghana OR Guatemala OR Guinea OR Guinea-Bissau OR Haiti OR Honduras OR India OR Indonesia OR Kenya OR Kiribati OR Korea, North OR Kyrgyzstan OR Laos OR Lesotho OR Liberia OR Madagascar OR Malawi OR Mali OR Mauritania OR Micronesia OR Mongolia OR Mozambique OR Myanmar OR Nepal OR Nicaragua OR Niger OR Nigeria OR Pakistan OR Papua New Guinea OR Philippines OR Rwanda OR Samoa OR Senegal OR Sierra Leone OR Solomon Islands OR Somalia OR South Sudan OR Sudan OR Syria OR Tajikistan OR Tanzania OR Timor-Leste OR Togo OR Tonga OR Turkmenistan OR Uganda OR Uzbekistan OR Vanuatu OR Venezuela OR Vietnam OR Yemen OR Zambia OR Zimbabwe)) |
| 4 | #1 AND #2 AND #3 |

| #3 | Search: "low-income countr*"[Title/Abstract] OR "middle-income countr*"[Title/Abstract] OR "developing countr*"[Title/Abstract] OR "low-resource countr*"[Title/Abstract] OR "resource-poor countr*"[Title/Abstract] OR "underdeveloped countr*"[Title/Abstract] OR "less developed countr*"[Title/Abstract] OR "global south"[Title/Abstract] OR "sub-Saharan Africa"[Title/Abstract] OR "Latin America"[Title/Abstract] OR "South Asia"[Title/Abstract] OR "Southeast Asia"[Title/Abstract] OR "Central America"[Title/Abstract] OR "Eastern Europe"[Title/Abstract] OR "Caribbean"[Title/Abstract] OR "Pacific Islands"[Title/Abstract] OR Afghanistan[Title/Abstract] OR Angola[Title/Abstract] OR Bangladesh[Title/Abstract] OR Benin[Title/Abstract] OR Bhutan[Title/Abstract] OR Bolivia[Title/Abstract] OR Botswana[Title/Abstract] OR Burkina Faso[Title/Abstract] OR Burundi[Title/Abstract] OR Cambodia[Title/Abstract] OR Cameroon[Title/Abstract] OR Central African Republic[Title/Abstract] OR Chad[Title/Abstract] OR Comoros[Title/Abstract] OR Congo[Title/Abstract] OR "Democratic Republic of the Congo"[Title/Abstract] OR Djibouti[Title/Abstract] OR Egypt[Title/Abstract] OR El Salvador[Title/Abstract] OR Eritrea[Title/Abstract] OR Eswatini[Title/Abstract] OR Ethiopia[Title/Abstract] OR Fiji[Title/Abstract] OR Gambia[Title/Abstract] OR Ghana[Title/Abstract] OR Guatemala[Title/Abstract] OR Guinea[Title/Abstract] OR Guinea-Bissau[Title/Abstract] OR Haiti[Title/Abstract] OR Honduras[Title/Abstract] OR India[Title/Abstract] OR Indonesia[Title/Abstract] OR Kenya[Title/Abstract] OR Kiribati[Title/Abstract] OR Korea, North[Title/Abstract] OR Kyrgyzstan[Title/Abstract] OR Laos[Title/Abstract] OR Lesotho[Title/Abstract] OR Liberia[Title/Abstract] OR Madagascar[Title/Abstract] OR Malawi[Title/Abstract] OR Mali[Title/Abstract] OR Mauritania[Title/Abstract] OR Micronesia[Title/Abstract] OR Mongolia[Title/Abstract] OR Mozambique[Title/Abstract] OR Myanmar[Title/Abstract] OR Nepal[Title/Abstract] OR Nicaragua[Title/Abstract] OR Niger[Title/Abstract] OR Nigeria[Title/Abstract] OR Pakistan[Title/Abstract] OR Papua New Guinea[Title/Abstract] OR Philippines[Title/Abstract] OR Rwanda[Title/Abstract] OR Samoa[Title/Abstract] OR Senegal[Title/Abstract] OR Sierra Leone[Title/Abstract] OR Solomon Islands[Title/Abstract] OR Somalia[Title/Abstract] OR South Sudan[Title/Abstract] OR Sudan[Title/Abstract] OR Syria[Title/Abstract] OR Tajikistan[Title/Abstract] OR Tanzania[Title/Abstract] OR Timor-Leste[Title/Abstract] OR Togo[Title/Abstract] OR Tonga[Title/Abstract] OR Turkmenistan[Title/Abstract] OR Uganda[Title/Abstract] OR Uzbekistan[Title/Abstract] OR Vanuatu[Title/Abstract] OR Venezuela[Title/Abstract] OR Vietnam[Title/Abstract] OR Yemen[Title/Abstract] OR Zambia[Title/Abstract] OR Zimbabwe[Title/Abstract] | [862,468](https://login.research4life.org/?term=longquery5bbe89d08a4757aed4d6&ac=no&sort=relevance) | 09:21:52 |
| --- | --- | --- | --- |
| #2 | Search: "women"[Title/Abstract] OR "pregnant women"[Title/Abstract] OR "mothers"[Title/Abstract] OR "females"[Title/Abstract] OR "prenatal" "perinatal"[Title/Abstract] OR "antenatal"[Title/Abstract] | [91,784](https://login.research4life.org/?term=%22women%22%5BTitle%2FAbstract%5D+OR+%22pregnant+women%22%5BTitle%2FAbstract%5D+OR+%22mothers%22%5BTitle%2FAbstract%5D+OR+%22females%22%5BTitle%2FAbstract%5D+OR+%22prenatal%22+%22perinatal%22%5BTitle%2FAbstract%5D+OR+%22antenatal%22%5BTitle%2FAbstract%5D&ac=no&sort=relevance) | 09:20:46 |
| #1 | Search**:** ("balanced energy protein supplements"[Title/Abstract] OR "energy protein supplements"[Title/Abstract] OR "protein-energy supplements"[Title/Abstract] OR "dietary protein supplements"[Title/Abstract] OR "balanced energy-protein"[Title/Abstract] OR "balanced energy and protein"[Title/Abstract] OR "balanced protein-energy"[Title/Abstract] OR "protein supplement"[Title/Abstract] OR "energy supplement"[Title/Abstract] OR "lipid supplement*"[Title/Abstract] OR "lipid nutrient*"[Title/Abstract] OR "lipid spread*"[Title/Abstract] OR "lipid paste*"[Title/Abstract] OR "nutributter"[Title/Abstract] OR "plumpy"[Title/Abstract] OR "fortified blended food"[Title/Abstract] OR "specialized nutritious food"[Title/Abstract] OR "ready to use food"[Title/Abstract] OR "corn soy* blend"[Title/Abstract] OR "wheat soya blend"[Title/Abstract] OR "super cereal"[Title/Abstract] OR "high energy biscuit"[Title/Abstract] OR "MAMTA"[Title/Abstract] OR "mumta"[Title/Abstract] OR "food assistance"[Title/Abstract] OR "food distribution"[Title/Abstract] OR "food specialized"[Title/Abstract] OR "food aid"[Title/Abstract] OR "food ration"[Title/Abstract] OR "food supplement*"[Title/Abstract] OR "food basket"[Title/Abstract] OR "supplement food"[Title/Abstract] OR "supplement feeding"[Title/Abstract] OR "oral supplements"[Title/Abstract] OR "fortified foods"[Title/Abstract] OR "powder mixes"[Title/Abstract] OR "protein bars"[Title/Abstract]) | [11,934](https://login.research4life.org/?term=%28%22balanced+energy+protein+supplements%22%5BTitle%2FAbstract%5D+OR+%22energy+protein+supplements%22%5BTitle%2FAbstract%5D+OR+%22protein-energy+supplements%22%5BTitle%2FAbstract%5D+OR+%22dietary+protein+supplements%22%5BTitle%2FAbstract%5D+OR+%22balanced+energy-protein%22%5BTitle%2FAbstract%5D+OR+%22balanced+energy+and+protein%22%5BTitle%2FAbstract%5D+OR+%22balanced+protein-energy%22%5BTitle%2FAbstract%5D+OR+%22protein+supplement%22%5BTitle%2FAbstract%5D+OR+%22energy+supplement%22%5BTitle%2FAbstract%5D+OR+%22lipid+supplement%2A%22%5BTitle%2FAbstract%5D+OR+%22lipid+nutrient%2A%22%5BTitle%2FAbstract%5D+OR+%22lipid+spread%2A%22%5BTitle%2FAbstract%5D+OR+%22lipid+paste%2A%22%5BTitle%2FAbstract%5D+OR+%22nutributter%22%5BTitle%2FAbstract%5D+OR+%22plumpy%22%5BTitle%2FAbstract%5D+OR+%22fortified+blended+food%22%5BTitle%2FAbstract%5D+OR+%22specialized+nutritious+food%22%5BTitle%2FAbstract%5D+OR+%22ready+to+use+food%22%5BTitle%2FAbstract%5D+OR+%22corn+soy%2A+blend%22%5BTitle%2FAbstract%5D+OR+%22wheat+soya+blend%22%5BTitle%2FAbstract%5D+OR+%22super+cereal%22%5BTitle%2FAbstract%5D+OR+%22high+energy+biscuit%22%5BTitle%2FAbstract%5D+OR+%22MAMTA%22%5BTitle%2FAbstract%5D+OR+%22mumta%22%5BTitle%2FAbstract%5D+OR+%22food+assistance%22%5BTitle%2FAbstract%5D+OR+%22food+distribution%22%5BTitle%2FAbstract%5D+OR+%22food+specialized%22%5BTitle%2FAbstract%5D+OR+%22food+aid%22%5BTitle%2FAbstract%5D+OR+%22food+ration%22%5BTitle%2FAbstract%5D+OR+%22food+supplement%2A%22%5BTitle%2FAbstract%5D+OR+%22food+basket%22%5BTitle%2FAbstract%5D+OR+%22supplement+food%22%5BTitle%2FAbstract%5D+OR+%22supplement+feeding%22%5BTitle%2FAbstract%5D+OR+%22oral+supplements%22%5BTitle%2FAbstract%5D+OR+%22fortified+foods%22%5BTitle%2FAbstract%5D+OR+%22powder+mixes%22%5BTitle%2FAbstract%5D+OR+%22protein+bars%22%5BTitle%2FAbstract%5D%29&ac=no&sort=relevance) | 09:18:07 |

| #1 | 'women'/exp OR 'women' OR 'pregnant women'/exp OR 'pregnant women' OR 'mothers'/exp OR 'mothers' OR  'females'/exp OR 'females' OR 'lactating  women'/exp OR 'lactating women' | 13,419,266 |
| --- | --- | --- |

**Cochrane Library (11/11/2024)**

| Search number | Search details | Hits |
| --- | --- | --- |
| #1 | ("women" OR "pregnant women" OR "mothers" OR "females" OR "lactating women" OR prenatal OR antenatal) | 262278 |
| #2 | ("balanced energy protein supplements" OR "energy protein supplements" OR "protein-energy supplements" OR "dietary protein supplements" OR "balanced energy-protein" OR "balanced energy and protein" OR "balanced protein-energy" OR "protein supplement" OR "energy supplement" OR "lipid supplement" OR "lipid nutrient" OR "lipid spread" OR "lipid paste" OR "nutributter" OR "plumpy" OR "fortified blended food" OR "specialized nutritious food" OR "ready to use food" OR "corn soy blend" OR "wheat soya blend" OR "super cereal" OR "high energy biscuit" OR "MAMTA" OR "mumta" OR "food assistance" OR "food distribution" OR "food specialized" OR "food aid" OR "food ration" OR "food supplement" OR "food basket" OR "supplement food" OR "supplement feeding" OR "oral supplements" OR "fortified foods" OR "powder mixes" OR "protein bars") | 2591 |
| #3 | ("low-income country" OR "middle-income country" OR "developing country" OR "low-resource country" OR "resource-poor country" OR "underdeveloped country" OR "less developed country" OR "global south" OR "sub-Saharan Africa" OR "Latin America" OR "South Asia" OR "Southeast Asia" OR "Central America" OR "Eastern Europe" OR "Caribbean" OR "Pacific Islands" OR Afghanistan OR Angola OR Bangladesh OR Benin OR Bhutan OR Bolivia OR Botswana OR Burkina Faso OR Burundi OR Cambodia OR Cameroon OR "Central African Republic" OR Chad OR Comoros OR Congo OR "Democratic Republic of the Congo" OR Djibouti OR Egypt OR "El Salvador" OR Eritrea OR Eswatini OR Ethiopia OR Fiji OR Gambia OR Ghana OR Guatemala OR Guinea OR "Guinea-Bissau" OR Haiti OR Honduras OR India OR Indonesia OR Kenya OR Kiribati OR "Korea, North" OR Kyrgyzstan OR Laos OR Lesotho OR Liberia OR Madagascar OR Malawi OR Mali OR Mauritania OR Micronesia OR Mongolia OR Mozambique OR Myanmar OR Nepal OR Nicaragua OR Niger OR Nigeria OR Pakistan OR "Papua New Guinea" OR Philippines OR Rwanda OR Samoa OR Senegal OR "Sierra Leone" OR "Solomon Islands" OR Somalia OR "South Sudan" OR Sudan OR Syria OR Tajikistan OR Tanzania OR "Timor-Leste" OR Togo OR Tonga OR Turkmenistan OR Uganda OR Uzbekistan OR Vanuatu OR Venezuela OR Vietnam OR Yemen OR Zambia OR Zimbabwe) | 95901 |
| #4 | #1 AND #2 AND #3 | 292 |
