# Supplementary material for: Delivering balanced energy and protein (BEP) supplements to pregnant and breastfeeding women in low- and middle-income countries: A scoping review
Source: PLOS Glob Public Health. 2026 Nov 2;6(11):e0007539. doi: 10.1371/journal.pgph.0007539 (PMC13726957; doi:10.1371/journal.pgph.0007539)
Supplement: Supplementary file 3 [file pgph.0007539.s003.docx]

**S2 Table:** Included Studies on Delivery Platforms for Iron and Folic Acid, and Multiple Micronutrient Supplements Among Pregnant and Breastfeeding Women

| **Author** | **Study design/data collection type** | **Country** | **Objective** | **Population at enrolment** | **Control** | **Intervention** | **Intervention form** | **Delivery mechanism** |
| --- | --- | --- | --- | --- | --- | --- | --- | --- |
| Siekmans et al., 2017 [1] | Formative research cross-sectional study – mixed methods | Afghanistan, Bangladesh, Indonesia, Ethiopia, Kenya, Nigeria, Senegal | To assess the enablers and barriers to improving IFA supplementation coverage and adherence. | Pregnant women, Health Care Providers and Social Influencers | N/A | IFA | N/A | Health facility |
| Palivela et al., 2021 [2] | Cross-sectional‑  study – quantitative methods | India | To assess the effect of direct monitoring of pregnant women by family members, counseling by health professionals, and other variables on adherence to IFAs in Puducherry, India. | A total of 257 pregnant women | N/A | IFA | Tablets | IFA was delivered at the health facility through the outpatient clinic with direct observed intake of IFA by family members at home. |
| Ahamed et al., 2018 [3] | Randomized controlled trial (RCT)- quantitative methods | India | To assess the effect of directly observed oral iron supplementation during pregnancy on iron status. | A total of 400 pregnant women | IFA tablets were supplemented without direct supervision | IFA | Tablets | Direct observation of supplement intake at home by CHW. |
| Kone et al., 2023 [4] | Cluster RCT- quantitative methods | Côte d’Ivoire | To assess the effectiveness of personal information sessions and personal information session plus home deliveries to increase coverage of IFA supplementation and Intermittent preventative malaria treatment, and their effectiveness on postpartum anemia and malaria infection. | A total of 767 pregnant women (age ≥15 years) in first or second trimester of pregnancy | IFA tablets were supplemented with routine ANC. | IFA | Tablets | Education delivery at home, education combined with home delivery of IFA supplements delivery (Intervention) and standard facility-based care (Control). |
| Ouedraogo at al., 2019 [5] | Quasi-experimental study design – quantitative methods | Niger | To assess the impact of the programmatic intervention on: (1) utilization of ANC, (2) adherence to daily IFA supplementation and (3) prevalence of adequate gestational weight gain and anemia among pregnant women in Zinder, Niger | A total of 2307 pregnant women from 68 clusters pregnant women | N/A | IFA | Tablets | Health facility plus BCC on pregnancy nutrition, IFA, early/regularly ANC, malaria prevention, danger sign recognition, and birth planning. |
| Seck and Jackson, 2008 [6] | RCT – mixed methods | Senegal | To determine and understand the specific factors that influence compliance with supplementation among pregnant women in Senegal. | A total of 221 pregnant women | IFA | IFA | Tablets | The intervention group received free IFA at a health facility whereas the control group received prescription to purchase IFA. |
| Zeng et al., 2009 [7] | Cluster-RCT – quantitative methods | China | To examine reports of side effects and rates of adherence to prenatal MMS in comparison with supplementation  with folic acid and with IFA and to describe inputs and costs associated with prenatal supplementation in China. | A total of 5828 pregnant Women | IFA, folic acid | MMS | Capsules | Initial supplements were provided at a health facility. The village doctor visited each woman at home to distribute additional supplements and collect  used blister strips. The number of remaining capsules was recorded. |
| Gebremichael and Welesamuel, 2020 [8] | Cross-sectional study – mixed methods | Ethiopia | To assess adherence to IFA supplement and its associated factors among antenatal care attending pregnant mothers in governmental health institutions of Adwa town. | A total of 623 pregnant women | N/A | IFA | Tablets | Health facility |
| Mbhenyane and Cherane, 2017 [9] | Exploratory cross-sectional study – mixed methods | South Africa | To investigate the factors that contribute to compliance to the consumption of IFA supplements by pregnant women. | A total of 57 pregnant women and 10 health workers | N/A | IFA | Tablets | Health facility |
| Paulino et al., 2005 [10] | Effectiveness study – mixed methods | Philippines | To describe the weekly IFA supplementation project activities and results on the intervention process. | A total of 744 women of reproductive age (339 pregnant and 409 non-pregnant) | N/A | IFA | Tablets | Health facility (supplementation was introduced within a social marketing framework and a social mobilization campaign) |
| Ridwan and Shafi, 2021 [11] | Cross-sectional study – mixed methods | Ethiopia | To assess the magnitude of adherence to IFA supplements and their associated factors among pregnant women attending ANC at public hospitals. | A total of 290 pregnant women | N/A | IFA | Tablets | Health facility |
| Sununtnasuk et al., 2015 [12] | Cross-sectional study – quantitative methods | Benin, Cambodia, Democratic Republic of Congo, Ethiopia, Ghana, Haiti, Honduras, Indonesia, Kenya, Liberia, Madagascar, Malawi, Mali, Nepal, Niger, Nigeria, Philippines, Rwanda, Senegal, Tanzania, Uganda, Zambia. | To identify where, within ANC programmes, pregnant women falter in obtaining and consuming an ideal minimum of 180 IFA tablets. | Pregnant women | N/A | IFA | Tablets | Health facility |
| Titaley and Dibley, 2015 [13] | Cross-sectional study – quantitative methods | Indonesia | To examine factors associated with non-use of antenatal iron/folic acid supplements in Indonesia. | Record from 26,591 deliveries, treated as 26,591 women (mothers) | N/A | IFA | Tablets | Health facility |
| Wana, 2020 [14] | Community-based cross-sectional study – mixed methods | Ethiopia | To identify the level and factors associated with utilization of prenatal iron supplementation. | A total of 411 pregnant women, 3 maternal child health department heads of health centers and four health extension workers. | N/A | IFA | Tablets | Health facility |
| Kamau et al., 2020 [15] | Pretest-posttest quasi-experimental design – quantitative methods | Kenya | To determine the effect of a community-based approach of IFA distribution on compliance and assess side-effects experienced and their mitigation by pregnant women. | A total of 340 pregnant women | IFA (from health facility) | IFA (from community health volunteers) | Tablets | Community health volunteers provided IFA, counselling and weekly follow-up to pregnant women (intervention group); control group followed standard practice from ANC at health facilities. |
| Shankar et al., 2009 [16] | RCT – quantitative methods | Indonesia | To examine how multiple-micronutrient supplementation during pregnancy reduced 90-day infant mortality by 20% compared with iron-folic acid. This analysis documents the programmatic impact of SUMMIT on health-seeking and early infant mortality resulting from community facilitators field activities. | 640 pregnant women who were enrolled in the Supplementation with Multiple Micronutrients Intervention (SUMMIT) | IFA | MMS | Powder | ANC at health facility with community facilitator incorporation to improve health-seeking practices and overall compliance with supplementation. |
| Nguyen et al., 2018 [17] | Cluster‐randomized program evaluation cluster‐randomized program evaluation – quantitative methods | Bangladesh | To examine how well the nutrition intervention was implemented and which elements of implementation explained intervention impact. | Pregnant women and 221 health workers, 494 health volunteers and 3000 women that recently delivered a baby | N/A | IFA | Tablets | Frontline workers (community health workers) conducted monthly home visits and supported ANC at health facilities |
| Risonar et al., 2007 [18] | Quasi experimental design (before and after study) – mixed methods | Philippines | To determine the effectiveness of the redesigned Fe supplementation delivery system (ISDS) for pregnant women in improving Fe status, reducing anemia prevalence, increasing compliance of pregnant women and in upgrading coverage by the health workers. | A total of 1180 pregnant women | N/A | IFA | Tablets | Home delivery of IFA every 15 days by CHWs and traditional birth attendant was a key feature of the redesigned ISDS, along with identifying pregnant women, monitoring supplement compliance, and using information, education and communications materials |
| Kim et al., 2023 [19] | Two arm cluster-randomized, non-masked trial with two cross-sectional surveys at baseline and endline ( program evaluation) – mixed methods | Burkina Faso | To assess the impacts of intensified nutrition interventions during ANC compared with standard ANC services on intervention coverage and maternal nutrition practices. | A total of 960 pregnant women and 1920 women with children 0–5 months of age per survey round from 40 clusters | The control areas received standard ANC services through the government health system. | IFA | Tablets | ANC services were provided by service providers (nurses and midwives) at government health and social promotion centers and community-based contacts through home visits and community meetings with pregnant and lactating women were conducted by community health agents;  The control areas received standard ANC services at. Health facility |
| Birhanu et al., 2018 [20] | Formative research - qualitative methods | Ethiopia | To examines Ethiopian women's perspective on ANC and IFA supplementation, identify barriers and facilitators to adherence, and inform the integration of calcium supplementation into ANC programs. | Pregnant women from rural districts in Oromia, Ethiopia, along with local health care providers and community volunteers. | N/A | IFA, calcium | Tablets | Health facility |
| Nguyen et al., 2017 [21] | Cluster-randomized design with cross-sectional surveys at baseline and endline – quantitative methods | Bangladesh | To evaluate the effect of providing nutrition-focused maternal nutrition and child health (MNCH) compared with standard MNCH (ANC with standard nutrition counseling) on coverage of nutrition interventions, maternal dietary diversity, micronutrient supplement intake, and early breastfeeding practices. | A total of 1000 pregnant and breastfeeding women from 300 clusters | N/A | IFA | Tablets | Counseling delivered by health workers and community volunteers in nutrition-focused MNCH; health workers conducted monthly home visits and ANC at a health facility with sessions covering diet plan demonstrations, free IFA and calcium with guidance, weight monitoring, rest counseling, and family engagement for food/supplement support. |
| Omotayo et al., 2018 [22] | Implementation study – mixed methods | Kenya | To describe the development of a comprehensive program for the integration of calcium supplementation into ANC platforms using the PIP approach and to determine whether calcium supplementation can be feasibly integrated with antenatal IFA supplementation in primary ANC. | A total of 16 health facilities and 990 women. | N/A | IFA, calcium | Tablets | Provided job aids (posters, counseling cards); trained CHWs on counseling, calcium and IFA supplementation, and use of behavior change tools (pill-taking calendars). Conducted four 6–8-hour training sessions on regimen, benefits, side effects, and counseling. Trained selected CHWs to mobilize pregnant women to ANC at health facility and reinforce messages. |
| Khan et al., 2005 [23] | Pre-post program evaluation study – quantitative methods | Vietnam | To promote a new preventive supplementation of IFA to WRA through social marketing and mobilization activities; and to assess the effectiveness of such a combined approach in improving knowledge, attitudes, and practices in the prevention and control of IDA. | A total of 863 pregnant & non-pregnant women | IFA | IFA | Tablets (120 mg iron and 3.5 mg folic acid) | Free home delivery  to pregnant women, but non pregnant women paid to purchase the supplements. |
| Clermont et al., 2018 [24] | Effectiveness randomized trial – mixed methods | Niger | To evaluate the acceptability and utilization of three nutritional supplements during pregnancy | Pregnant women (84 interviews and 17 FGDs discussion in phase 1; 39 interviews and 12 FGDs in phase 2), household members, and study staff | IFA | MMS | Tablets | Home delivery |
| Nguyen et al., 2021 [25] | Cluster-randomized design with cross-sectional surveys at baseline and endline – quantitative methods | India | To compare nutrition-intensified ANC with standard ANC on coverage of nutrition interventions and maternal nutrition practices. | A total of 660 pregnant women, 1800 recently delivered women, and 400 pregnant women in a longitudinal follow-up | IFA | IFA | N/A Visit | ANC at health facility and home visits |
| Sahu et al., 2025 [26] | Cross-sectional study - mixed methods | India | To assess the gap in services and the current implementation status of the Village Health and Nutrition Day services | Pregnant and lactating women (beneficiaries) | N/A | IFA and calcium | Tablets | ANC services provided by CHW at community centers (Anganwadi Centers) |
| IFA = Iron and Folic Acid; MMS = Multiple Micronutrient Supplement; ANC = Antenatal Care; BCC = Behaviors Change Communication; CHW = Community Health Worker; MNCH = Maternal, Newborn and Child Health; RCT = Randomized Controlled Trial; ISDS = Iron Supplementation Delivery System; PIP = Program Implementation Pathway; LNS = Lipid-based Nutrient Supplement; N/A = Non-applicable | | | | | | | | |

References

1.	Siekmans K, Roche M, Kung’u JK, Desrochers RE, De-Regil LM. Barriers and enablers for iron folic acid (IFA) supplementation in pregnant women. Matern Child Nutr. 2018;14 Suppl 5: e12532. doi:10.1111/mcn.12532

2.	Palivela D, Shehnaz SI, Chaturvedula L. Effect of direct monitoring by family members and counseling by health professionals on iron-folic acid supplementation: A cross-sectional study among pregnant women in Puducherry, India. J Fam Community Med. 2021;28: 85–93. doi:10.4103/jfcm.JFCM_445_20

3.	Ahamed F, Yadav K, Kant S, Saxena R, Bairwa M, Pandav CS. Effect of directly observed oral iron supplementation during pregnancy on iron status in a rural population in Haryana: A randomized controlled trial. Indian J Public Health. 2018;62: 287–293. doi:10.4103/IJPH.IJPH_313_17

4.	Koné S, Probst-Hensch N, Dao D, Utzinger J, Fink G. Improving coverage of antenatal iron and folic acid supplementation and malaria prophylaxis through targeted information and home deliveries in Côte d’Ivoire: a cluster randomised controlled trial. BMJ Glob Health. 2023;8. doi:10.1136/BMJGH-2022-010934

5.	Ouedraogo CT, Wessells TR, Young RR, Bamba IF, Faye MT, Banda N, et al. The mixed effects of a package of multilevel interventions on the health and care of pregnant women in Zinder, Niger. BMJ Glob Health. 2019;4: 1200–1200.

6.	Seck BC, Jackson RT. Determinants of compliance with iron supplementation among pregnant women in Senegal. PUBLIC Health Nutr. 2008;11: 596–605.

7.	Zeng L, Yan H, Cheng Y, Dang S, Dibley MJ. Adherence and costs of micronutrient supplementation in pregnancy in a double-blind, randomized, controlled trial in rural western China. Food Nutr Bull. 2009;30: S480-487. doi:10.1177/15648265090304S402

8.	Gebremichael TG, Welesamuel TG. Adherence to iron-folic acid supplement and associated factors among antenatal care attending pregnant mothers in governmental health institutions of Adwa town, Tigray, Ethiopia: Cross-sectional study. PloS One. 2020;15: e0227090–e0227090. doi:10.1371/journal.pone.0227090

9.	Mbhenyane X, Cherane M. Compliance with the consumption of iron and folate supplements by pregnant women in Mafikeng local municipality, North West province, South Africa. Afr Health Sci. 2017;17: 657–670. doi:10.4314/ahs.v17i3.8

10.	Paulino LS, Angeles-Agdeppa I, Etorma UMM, Ramos AC, Cavalli-Sforza T. Weekly iron-folic acid supplementation to improve iron status and prevent pregnancy anemia in Filipino women of reproductive age: the Philippine experience through government and private partnership. Nutr Rev. 2005;63: S109-115. doi:10.1301/nr.2005.dec.s109-s115

11.	Ridwan N, Shafi A. Adherence to iron folate supplementation and associated factors among pregnant women attending antenatal care at public hospitals in Jigjiga Town, Somali Region, Ethiopia 2020. Pan Afr Med J. 2021;40: 196–196. doi:10.11604/pamj.2021.40.196.27958

12.	Sununtnasuk C, D’Agostino A, Fiedler JL. Iron+folic acid distribution and consumption through antenatal care: identifying barriers across countries. Public Health Nutr. 2016;19: 732–742. doi:10.1017/S1368980015001652

13.	Titaley CR, Dibley MJ. Factors associated with not using antenatal iron/folic acid supplements in Indonesia: the 2002/2003 and 2007 Indonesia Demographic and Health Survey. Asia Pac J Clin Nutr. 2015;24: 162–176. doi:10.6133/apjcn.2015.24.1.10

14.	Wana EW. Predictors of prenatal iron folic acid supplement utilization in Wolaita, South Ethiopia: a community based cross-sectional study (quantitative and qualitative approach). BMC Pregnancy Childbirth. 2020;20: 243. doi:10.1186/s12884-020-02883-2

15.	Kamau MW, Kimani ST, Mirie W, Mugoya IK. Effect of a community-based approach of iron and folic acid supplementation on compliance by pregnant women in Kiambu County, Kenya: A quasi-experimental study. PLoS ONE. 2020;15: e0227351. doi:10.1371/journal.pone.0227351

16.	Shankar AV, Asrilla Z, Kadha JK, Sebayang S, Apriatni M, Sulastri A, et al. Programmatic effects of a large-scale multiple-micronutrient supplementation trial in Indonesia: Using community facilitators as intermediaries for behavior change. FOOD Nutr Bull. 2009;30: S207–S214. doi:10.1177/15648265090302S204

17.	Nguyen PH, Frongillo EA, Sanghvi T, Kim SS, Alayon S, Tran LM, et al. Importance of coverage and quality for impact of nutrition interventions delivered through an existing health programme in Bangladesh. Matern Child Nutr. 2018;14: 12613–12613.

18.	Risonar MGD, Rayco-Solon P, Tengco LW, Sarol Jr. JN, Paulino LS, Solon FS. Effectiveness of a redesigned iron supplementation delivery system for pregnant women in Negros Occidental, Philippines. PUBLIC Health Nutr. 2009;12: 932–940. doi:10.1017/S1368980008003418

19.	Kim SS, Zagre RR, Ouedraogo CT, Sununtnasuk C, Ganaba R, Zafimanjaka MG, et al. Intensified Nutrition Interventions in Antenatal Care Services Increased Consumption of Iron and Folic Acid Supplements and Early Breastfeeding Practices in Burkina Faso: Results of a Cluster-Randomized Program Evaluation. J Nutr. 2023;153: 3058–3067.

20.	Birhanu Z, Chapleau GM, Ortolano SE, Mamo G, Martin SL, Dickin KL. Ethiopian women’s perspectives on antenatal care and iron-folic acid supplementation: Insights for translating global antenatal calcium guidelines into practice. Matern Child Nutr. 2018;14: 12424–12424.

21.	Nguyen PH, Kim SS, Sanghvi T, Mahmud Z, Tran LM, Shabnam S, et al. Integrating Nutrition Interventions into an Existing Maternal, Neonatal, and Child Health Program Increased Maternal Dietary Diversity, Micronutrient Intake, and Exclusive Breastfeeding Practices in Bangladesh: Results of a Cluster-Randomized Program Eval. J Nutr. 2017;147: 2326–2337.

22.	Omotayo MO, Dickin KL, Pelletier DL, Martin SL, Kung’u JK, Stoltzfus RJ. Feasibility of integrating calcium and iron-folate supplementation to prevent preeclampsia and anemia in pregnancy in primary healthcare facilities in Kenya. Matern Child Nutr. 2018;14: 12437–12437.

23.	Khan NC, Thanh HTK, Berger J, Hoa PT, Quang ND, Smitasiri S, et al. Community mobilization and social marketing to promote weekly iron-folic acid supplementation: A new approach toward controlling anemia among women of reproductive age in Vietnam. Nutr Rev. 2005;63: S87–S94.

24.	Clermont A, Kodish SR, Seck AM, Salifou A, Rosen J, Grais RF, et al. Acceptability and Utilization of Three Nutritional Supplements during Pregnancy: Findings from a Longitudinal, Mixed-Methods Study in Niger. Nutrients. 2018;10. doi:10.3390/NU10081073

25.	Nguyen PH, Kachwaha S, Tran LM, Avula R, Young MF, Ghosh S, et al. Strengthening Nutrition Interventions in Antenatal Care Services Affects Dietary Intake, Micronutrient Intake, Gestational Weight Gain, and Breastfeeding in Uttar Pradesh, India: Results of a Cluster-Randomized Program Evaluation. J Nutr. 2021;151: 2282–2295.

26.	Sahu DP, Preeti SP, Singh AK, Bhatia V. Service Gap Assessment of Village Health and Nutrition Day Services in a Rural Block of Eastern Odisha: A Mixed-method Study. Indian J Community Med. 2025;50: S35–S40. doi:10.4103/ijcm.ijcm_293_23
