# Supplementary material for: Delivering balanced energy and protein (BEP) supplements to pregnant and breastfeeding women in low- and middle-income countries: A scoping review
Source: PLOS Glob Public Health. 2026 Nov 2;6(11):e0007539. doi: 10.1371/journal.pgph.0007539 (PMC13726957; doi:10.1371/journal.pgph.0007539)
Supplement: Supplementary file 4 [file pgph.0007539.s004.docx]

S1 Appendix: Indirect Evidence on IFA and MMS Supplementation

**Methods:**

Iron and folic acid (IFA) is a prenatal supplement containing iron and folic acid. Multiple micronutrient supplements (MMS) was defined according to World Health Organization (WHO): a prenatal supplement containing at least three micronutrients in addition to iron and folic acid [1].

**Results**:

A total of 26 studies evaluated supplementation with IFA or MMS without any balanced energy protein (BEP) supplementation [2–27] as shown in **S4 Table**. Twenty-one studies examined IFA supplementation alone across low-and middle-income countries (LMICs), particularly in South Asia and sub-Saharan Africa. Three additional studies combined IFA with calcium supplementation and three studies focused on MMS. Overall, this indirect evidence showed mixed findings regarding antenatal care (ANC) health facility delivery, home delivery (including direct observation of intake), and market delivery approaches.

Community feeding centers of IFA and MMS supplements

Only one study evaluated community feeding centers, reporting delivery of IFA and calcium by community health workers through the Village Health and Nutrition Day service in India [22].

Health facility delivery of IFA and MMS supplements

The provision of micronutrient supplements via ANC services at health facilities was the most reported delivery platform, occurring in 17 of the 25 studies [3,4,6–13,17–19,23–26]. However, there are enhancements to some of the ANC service-based approaches reported, such as the incorporation of directly observed supplement intake by family members [4], behavior change communication or counseling [6,13,14,17,19,20,27], integration of social marketing campaigns [11], and subsequent home visits post initial supplement provision at the health facility [5,8,16,19]. Across diverse contexts, the evidence shows a consistently positive trend: supplement delivery strategies that extend beyond routine facility-based care, such as home delivery or community mobilization, substantially improve supplement adherence [5,13,16,19,27,28]. A study conducted in Côte d’Ivoire showed that adding home delivery of IFA (alongside intermittent preventative treatment for malaria) to information sessions markedly improved IFA adherence [5]. In Bangladesh, nutrition-focused maternal and child health services at health facilities supplemented with monthly home visits, provision of free IFA and calcium, and family engagement increased IFA intake (+46 tablets), calcium intake (+50 tablets), and dietary diversity (+1.6 food groups; meeting minimum diversity of ≥5 groups, 30%) [19]. In China, adherence was very high under a program combining free MMS with biweekly home resupply by village doctors, with over 90% of participants taking their supplements on at least 165 days, and ~40% of participants reaching 180 capsules [28]. In the Philippines, a redesigned system using village health workers and traditional-birth-attendant-led mapping, counseling, and 15-day home delivery of IFA, increased prenatal visits (from 0.9 to 1.66 per trimester); participants were four times more likely to take IFA and ~17 times more likely to be monitored during pregnancy than health facility-only delivery [16]. Findings from a multi-country study that included 22 African countries further underscore the need for improved ANC delivery, highlighting gaps in micronutrient supplement delivery at four key points: ANC attendance, micronutrient supplement receipt, intake of at least one micronutrient-containing supplement, and completion of the recommended micronutrient supplement dose [23]. Similarly, a multi-country formative study conducted in Afghanistan, Bangladesh, Indonesia, Ethiopia, Kenya, Nigeria, and Senegal identified various barriers to accessing IFA at ANC clinics [26]. In Senegal, the high cost of ANC clinics was reported as a barrier, while in Ethiopia, limited human resources hindered service provision. Bangladesh faced challenges due to a lack of resources. In Afghanistan, despite accessible health facilities with adequate staffing and supplies, long waiting times were reported as a key challenge [26].

Home delivery of IFA and MMS supplements

Home delivery of micronutrients with incorporating ANC service delivery was reported in four studies [2,5,26,29]. Pregnant women from Niger reported that home delivery of MMS and IFA was “helpful and convenient” [29]. An RCT conducted in Côte d’Ivoire that compared delivery platforms of IFA found that adherence to IFA was higher in pregnant women who received monthly IFA home deliveries (coupled with ANC) compared to those who received ANC clinic deliveries [5]. Similarly, in an RCT in India, direct observation of IFA intake at home improved participants’ iron status compared with ANC delivery alone; however, it was unclear whether IFA was delivered at home [2]. A multi-country study conducted in Afghanistan, Bangladesh, Indonesia, Ethiopia, Kenya, Nigeria, and Senegal reported that home delivery of IFA using community health workers was rare compared to health facility delivery [26]; in these countries, the role of community health workers was often restricted to providing nutrition counseling. Notably, in Afghanistan, community health workers were perceived as "illiterate and unknowledgeable or not doctors" by some pregnant women. Within the same study, pregnant women viewed the provision of supplements by health workers at community health posts as an enabler for accessing IFA supplementation, due to the proximity of ANC clinics at community health posts compared to health centers [26].

Market delivery of IFA and MMS supplements

One study explored the incorporation of the market approach and reported on the free distribution of products to pregnant women in Vietnam [21]. A social marketing and mobilization strategy for weekly IFA, involving free distribution to pregnant women via commune health stations, achieved nearly universal coverage [21].

References

1.	WHO antenatal care recommendations for a positive pregnancy experience: Nutritional interventions update: Multiple micronutrient supplements during pregnancy. Geneva: World Health Organization; 2020. Available: http://www.ncbi.nlm.nih.gov/books/NBK560384/

2.	Ahamed F, Yadav K, Kant S, Saxena R, Bairwa M, Pandav CS. Effect of directly observed oral iron supplementation during pregnancy on iron status in a rural population in Haryana: A randomized controlled trial. Indian J Public Health. 2018;62: 287–293. doi:10.4103/IJPH.IJPH_313_17

3.	Nguyen PH, Kachwaha S, Tran LM, Avula R, Young MF, Ghosh S, et al. Strengthening Nutrition Interventions in Antenatal Care Services Affects Dietary Intake, Micronutrient Intake, Gestational Weight Gain, and Breastfeeding in Uttar Pradesh, India: Results of a Cluster-Randomized Program Evaluation. J Nutr. 2021;151: 2282–2295.

4.	Palivela D, Shehnaz SI, Chaturvedula L. Effect of direct monitoring by family members and counseling by health professionals on iron-folic acid supplementation: A cross-sectional study among pregnant women in Puducherry, India. J Fam Community Med. 2021;28: 85–93. doi:10.4103/jfcm.JFCM_445_20

5.	Koné S, Probst-Hensch N, Dao D, Utzinger J, Fink G. Improving coverage of antenatal iron and folic acid supplementation and malaria prophylaxis through targeted information and home deliveries in Côte d’Ivoire: a cluster randomised controlled trial. BMJ Glob Health. 2023;8. doi:10.1136/BMJGH-2022-010934

6.	Ouedraogo CT, Wessells TR, Young RR, Bamba IF, Faye MT, Banda N, et al. The mixed effects of a package of multilevel interventions on the health and care of pregnant women in Zinder, Niger. BMJ Glob Health. 2019;4: 1200–1200.

7.	Seck BC, Jackson RT. Determinants of compliance with iron supplementation among pregnant women in Senegal. PUBLIC Health Nutr. 2008;11: 596–605.

8.	Zeng L, Yan H, Cheng Y, Dang S, Dibley MJ. Adherence and costs of micronutrient supplementation in pregnancy in a double-blind, randomized, controlled trial in rural western China. FOOD Nutr Bull. 2009;30: S480–S487. doi:10.1177/15648265090304S402

9.	Gebremichael TG, Welesamuel TG. Adherence to iron-folic acid supplement and associated factors among antenatal care attending pregnant mothers in governmental health institutions of Adwa town, Tigray, Ethiopia: Cross-sectional study. PloS One. 2020;15: e0227090–e0227090. doi:10.1371/journal.pone.0227090

10.	Mbhenyane X, Cherane M. Compliance with the consumption of iron and folate supplements by pregnant women in Mafikeng local municipality, North West province, South Africa. Afr Health Sci. 2017;17: 657–670. doi:10.4314/ahs.v17i3.8

11.	Paulino LS, Angeles-Agdepp I, Etorma UMM, Ramos AC, Cavalli-Sforza T. Weekly iron-folic acid supplementation to improve iron status and prevent pregnancy anemia in Filipino women of reproductive age: The Philippine experience through government and private partnership. Nutr Rev. 2005;63: S109–S115.

12.	Ridwan N, Shafi A. Adherence to iron folate supplementation and associated factors among pregnant women attending antenatal care at public hospitals in Jigjiga Town, Somali Region, Ethiopia 2020. Pan Afr Med J. 2021;40: 196–196. doi:10.11604/pamj.2021.40.196.27958

13.	Shankar AV, Asrilla Z, Kadha JK, Sebayang S, Apriatni M, Sulastri A, et al. Programmatic effects of a large-scale multiple-micronutrient supplementation trial in Indonesia: Using community facilitators as intermediaries for behavior change. FOOD Nutr Bull. 2009;30: S207–S214. doi:10.1177/15648265090302S204

14.	Kamau MW, Kimani ST, Mirie W, Mugoya IK. Effect of a community-based approach of iron and folic acid supplementation on compliance by pregnant women in Kiambu County, Kenya: A quasi-experimental study. PloS One. 2020;15: e0227351–e0227351. doi:10.1371/journal.pone.0227351

15.	Nguyen PH, Frongillo EA, Sanghvi T, Kim SS, Alayon S, Tran LM, et al. Importance of coverage and quality for impact of nutrition interventions delivered through an existing health programme in Bangladesh. Matern Child Nutr. 2018;14: 12613–12613.

16.	Risonar MGD, Rayco-Solon P, Tengco LW, Sarol Jr. JN, Paulino LS, Solon FS. Effectiveness of a redesigned iron supplementation delivery system for pregnant women in Negros Occidental, Philippines. PUBLIC Health Nutr. 2009;12: 932–940. doi:10.1017/S1368980008003418

17.	Kim SS, Zagre RR, Ouedraogo CT, Sununtnasuk C, Ganaba R, Zafimanjaka MG, et al. Intensified Nutrition Interventions in Antenatal Care Services Increased Consumption of Iron and Folic Acid Supplements and Early Breastfeeding Practices in Burkina Faso: Results of a Cluster-Randomized Program Evaluation. J Nutr. 2023;153: 3058–3067.

18.	Birhanu Z, Chapleau GM, Ortolano SE, Mamo G, Martin SL, Dickin KL. Ethiopian women’s perspectives on antenatal care and iron-folic acid supplementation: Insights for translating global antenatal calcium guidelines into practice. Matern Child Nutr. 2018;14: 12424–12424.

19.	Nguyen PH, Kim SS, Sanghvi T, Mahmud Z, Tran LM, Shabnam S, et al. Integrating Nutrition Interventions into an Existing Maternal, Neonatal, and Child Health Program Increased Maternal Dietary Diversity, Micronutrient Intake, and Exclusive Breastfeeding Practices in Bangladesh: Results of a Cluster-Randomized Program Eval. J Nutr. 2017;147: 2326–2337.

20.	Omotayo MO, Dickin KL, Pelletier DL, Martin SL, Kung’u JK, Stoltzfus RJ. Feasibility of integrating calcium and iron-folate supplementation to prevent preeclampsia and anemia in pregnancy in primary healthcare facilities in Kenya. Matern Child Nutr. 2018;14: 12437–12437.

21.	Khan NC, Thanh HTK, Berger J, Hoa PT, Quang ND, Smitasiri S, et al. Community mobilization and social marketing to promote weekly iron-folic acid supplementation: A new approach toward controlling anemia among women of reproductive age in Vietnam. Nutr Rev. 2005;63: S87–S94.

22.	Sahu DP, Preeti SP, Singh AK, Bhatia V. Service Gap Assessment of Village Health and Nutrition Day Services in a Rural Block of Eastern Odisha: A Mixed-method Study. Indian J Community Med. 2025;50: S35–S40. doi:10.4103/ijcm.ijcm_293_23

23.	Sununtnasuk C, D’Agostino A, Fiedler JL. Iron+folic acid distribution and consumption through antenatal care: identifying barriers across countries. Public Health Nutr. 2016;19: 732–742. doi:10.1017/S1368980015001652

24.	Titaley CR, Dibley MJ. Factors associated with not using antenatal iron/folic acid supplements in Indonesia: the 2002/2003 and 2007 Indonesia Demographic and Health Survey. Asia Pac J Clin Nutr. 2015;24: 162–176. doi:10.6133/apjcn.2015.24.1.10

25.	Wana EW. Predictors of prenatal iron folic acid supplement utilization in Wolaita, South Ethiopia: a community based cross-sectional study (quantitative and qualitative approach). BMC Pregnancy Childbirth. 2020;20: 243. doi:10.1186/s12884-020-02883-2

26.	Siekmans K, Roche M, Kung’u JK, Desrochers RE, De-Regil LM. Barriers and enablers for iron folic acid (IFA) supplementation in pregnant women. Matern Child Nutr. 2018;14 Suppl 5: e12532. doi:10.1111/mcn.12532

27.	Hess SY, Ouédraogo CT, Bamba IF, Wessells KR, Keith N, Faye T, et al. Using formative research to promote antenatal care attendance and iron folic acid supplementation in Zinder, Niger. Matern Child Nutr. 2018;14: e12525. doi:10.1111/mcn.12525

28.	Zeng L, Yan H, Cheng Y, Dang S, Dibley MJ. Adherence and costs of micronutrient supplementation in pregnancy in a double-blind, randomized, controlled trial in rural western China. Food Nutr Bull. 2009;30: S480-487. doi:10.1177/15648265090304S402

29.	Clermont A, Kodish SR, Seck AM, Salifou A, Rosen J, Grais RF, et al. Acceptability and Utilization of Three Nutritional Supplements during Pregnancy: Findings from a Longitudinal, Mixed-Methods Study in Niger. Nutrients. 2018;10. doi:10.3390/NU10081073
